# Bringt die Abdeckung des ovalen Fensters zusätzlich zum runden Fenster bei der Reservetherapie der akuten idiopathischen Ertaubung einen Vorteil?

**DOI:** 10.1007/s00106-020-00903-3

**Published:** 2020-07-29

**Authors:** V. M. Hofmann, U. Schoenfeld, M. Jagielski, A. Pudszuhn

**Affiliations:** Klinik für Hals‑, Nasen- und Ohrenheilkunde, Charité – Universitätsmedizin Berlin, Campus Benjamin Franklin, Hindenburgdamm 30, 12200 Berlin, Deutschland

**Keywords:** Plötzliche Ertaubung, Perilymphfistel, Tympanoskopie, Rundfenstermembran, Hörschwelle, Acute deafness, Perilymph fistula, Tympanotomy, Round window, Hearing threshold

## Abstract

**Hintergrund:**

Bei plötzlich aufgetretener einseitiger hochgradiger sensorineuraler Hörstörung bzw. Ertaubung können Patienten nach frustraner intravenöser Glukokortikoidtherapie einer explorativen Tympanotomie mit Abdeckung des runden und/oder ovalen Fensters (RF und/oder OF) wegen des Verdachts einer Rundfenstermembranruptur mit Perilymphfistel (PLF) oder einer Fissula ante fenestram (FAF) behandelt werden. Die Studie untersucht, ob die zusätzliche Abdeckung des ovalen Fensters im Vergleich zur alleinigen Abdeckung des runden Fensters einen zusätzlichen Hörgewinn bringt.

**Methodik:**

Die retrospektive Studie untersucht 54 Patienten mit akuter Ertaubung und durchgeführter Tympanoskopie, bei denen Audiogramme von 3 Zeitpunkten (präoperativ, nach Detamponade sowie mindestens 3–6 Monate postoperativ) vorlagen. Bei 28 Patienten erfolgte neben der Rundfensterabdeckung die zusätzliche Abdeckung des ovalen Fensters.

**Ergebnis:**

Eine intraoperativ sichtbare PFL bzw. FAF wurde in keinem Operationsbericht beschrieben. Die Hörschwellen waren im frühen postoperativen Zeitraum bereits hoch signifikant geringer und verbesserten sich im weiteren Verlauf. Zwischen den Subgruppen RF und RF + OF zeigte sich in beiden postoperativen Zeiträumen kein signifikanter Unterschied. Bei 65 % (Kriterien nach Kanzaki) und 74 % (Kriterien nach Siegel) der Patienten konnte postoperativ eine partielle bzw. komplette Hörverbesserung nachgewiesen werden. Beim Vergleich der Patientengruppen mit bzw. ohne postoperative Hörverbesserung zeigten sich keine statistischen Unterschiede bezüglich Geschlecht, Alter, Nebendiagnosen oder Latenz der Operation bis zum Symptombeginn.

**Schlussfolgerung:**

In dieser Studie zeigte die zusätzliche Abdeckung des OF keine signifikante Hörverbesserung. Die postoperative Hörverbesserung entspricht den publizierten Spontanremissionsraten. Nur eine prospektive multizentrische Studie mit Kontrollgruppe kann den Stellenwert der Tympanoskopie und mit Rund- bzw. ovaler Fensterabdeckung bei einer akuten hochgradigen sensorineuralen Schwerhörigkeit vor dem Hintergrund der Spontanremissionen klären.

## Abstract (en)

**Background:**

Following sudden unilateral deafness or severe sensorineural hearing loss, patients with unsuccessful intravenous steroid therapy can be treated with explorative tympanotomy with sealing of the round (RW) and/or oval window (OW), due to suspected rupture of the RW with perilymph fistula (PLF) or a fissula ante fenestram (FAF). This study investigated whether additional sealing of the oval window (RW+OW) achieved an improved hearing benefit as compared to sealing of the round window only (RW) .

**Methods:**

This retrospective study investigated 54 patients with acute profound hearing loss who underwent tympanoscopy. Audiometric examinations were performed preoperatively and at two postoperative intervals (1 month and 3–6 months after surgery). In 28 patients, the OW was sealed in addition to the RW.

**Results:**

No intraoperatively visible PLF or FAF were reported. Hearing thresholds were significantly reduced in the early postoperative follow-up period and further improvement was observed 3–6 months later. No significant differences between the RW and RW+OW subgroups were seen at either follow-up timepoint. In 65% (Kanzaki criteria) and 74% (Siegel criteria) of patients, partial or complete postoperative hearing improvement was observed. Upon comparing the groups of patients with and without hearing improvement, no statistical significance was found in terms of gender, age, secondary diagnoses, or latency period between symptom onset and surgery.

**Conclusion:**

Additional sealing of the OW did not lead to significantly better postoperative hearing thresholds. In general, postoperative hearing improvement corresponds to published spontaneous remission rates.

Eine akut aufgetretene, meist einseitige Schallempfindungsschwerhörigkeit unklarer Genese wird als Hörsturz bezeichnet [2]. Hörstürze treten in unterschiedlichen Schweregraden bis hin zur vollständigen Ertaubung auf. Sie sind mit einer Inzidenz von 160–400/100.000/Jahr ein relativ häufiges Krankheitsbild [5, 15, 37]. Die genauen Inzidenzen sind aufgrund fehlender epidemiologischer Daten und der relativ häufigen Spontanremission (unter anderem noch vor dem Arztbesuch) nicht eindeutig zu eruieren. Die WHO-Klassifikation unterscheidet die hochgradige Schwerhörigkeit („severe hearing loss“; mittlerer Hörverlust 61–80 dB) von der Ertaubung („profound hearing loss“; mittlerer Hörverlust >80 dB) [24]. Für den mittleren Hörverlust werden die tonaudiometrischen Messwerte bei den Frequenzen 500 Hz, 1000 Hz, 2000 Hz und 4000 Hz gemittelt („four pure tone average“, 4‑PTA) [24].

Um einen „Hörsturz“ handelt es sich dann, wenn die Ursache der plötzlich aufgetretenen hochgradigen Schallempfindungsschwerhörigkeit bzw. Ertaubung durch Anamnese, klinische Untersuchung, Hörprüfungen, periphere Gleichgewichtsuntersuchungen und Bildgebung ungeklärt bleibt [28]. Häufig beruhen akute Hörminderungen auf vaskulären, metabolischen, (auto)immunologischen, infektiösen, neoplastischen, chemisch-toxischen oder entzündlichen Ursachen [28]. Als weitere Ursache der akuten Ertaubung wird eine Ruptur des RF [28] bzw. die Fissula ante fenestram (FAF) [39] diskutiert.

Die tatsächliche Existenz einer Rundfenstermembranruptur als häufige Ursache ist umstritten. Ein akutes oder auch zurückliegendes Trauma wird diskutiert, in den meisten Fällen ist die Patientenanamnese eines ertaubten Patienten bezüglich eines Traumas aber unauffällig [37]. Die Ursache einer FAF könnte in einer beeinträchtigen knöchernen Remodellierung der Cochlea in der Wachstumsphase liegen [39].

Außer der subjektiven konventionellen Ton- und Sprachaudiometrie existieren keine objektiven Testverfahren, die eine Rundfenstermembranruptur mit Perilymphfistel (PLF) eindeutig nachweisen. Eine FAF kann möglicherweise in einer hochauflösenden Computertomographie (CT) des Felsenbeins an einer kleinen, anterior der ovalen Nische sichtbaren knöchernen Spalte erkannt werden [39]. Diagnostisch lässt sich die Ursache der plötzlichen Ertaubung in den meisten Fällen nicht klären.

Therapeutisch erfolgt bei einer akuten hochgradigen Schwerhörigkeit bzw. plötzlichen Ertaubung in den meisten Kliniken in Analogie zur weniger ausgeprägten akuten Hörminderung zunächst eine, den Leitlinien entsprechende, konservative Behandlung mit einem intravenös applizierten Glukokortikoidschema (z. B. Prednisolon) [2]. Kommt es im Verlauf zu keiner Hörverbesserung, wird der Patient (meist als Sekundär- oder Tertiärtherapie) über die Möglichkeit einer explorativen Tympanotomie aufgeklärt. Wird bei diesem Eingriff der Verdacht auf eine Rundfenstermembranruptur mit PLF oder FAF gestellt, erfolgt intraoperativ die Versorgung des runden Fensters oder der FAF mit in Dexamethasonphosphat getränktem Bindegewebe und dexamethasonphosphathaltigem Gelita-Tampon [18].

Auch intraoperativ ist der eindeutige Nachweis einer PLF dadurch erschwert, dass die Rundfenstermembran aufgrund eines etwas weiter lateral liegenden membranösen Häutchens häufig nicht vollständig einsehbar ist [29]. Eine FAF sei nach vorsichtigem Absaugen einfach sichtbar und behandelbar [39].

Zeigt sich posttherapeutisch eine Hörverbesserung, ist auch das aufgrund der hohen Spontanremissionsrate kein Beweis für eine Rundfenstermembranruptur oder eine FAF.

Eine sich nach der Operation einstellende Hörverbesserung könnte auch Folge der direkten Applikation von Dexamethasonphosphat im Bereich der Rundfensternische sein, sodass weniger die Abdichtung des runden Fensters, sondern, in Analogie zur intratympanalen Dexamethasonphosphatapplikation, das als Depot applizierte Medikament die therapeutisch wirksame Maßnahme darstellt [32].

Trotz der Existenz zahlreicher Studien ist der Nutzen des operativen Vorgehens nicht erbracht [9]. Andererseits sind bei der Durchführung einer explorativen Tympanotomie mit Rundfenstermembranabdeckung bzw. Fistelabdeckung auch keine schweren Komplikationen zu erwarten, die ärztlicherseits zur kategorischen Ablehnung des Eingriffs führen. Der Eingriff mit einer Dauer von ca. 30–45 min kann in Intubationsnarkose oder in Lokalanästhesie durchgeführt werden. Auch wenn sich intraoperativ keine FAF oder PLF zeigen, erfolgt meistens dennoch die intratympanale Dexamethasonapplikation [9, 12, 18].

Sollte die Medikamentenapplikation als Depot in den Nischen [28] den Haupteffekt darstellen, könnte die zusätzliche Abdeckung bzw. Applikation von Dexamethasonphosphat am ovalen Fenster einen zusätzlichen Nutzen bringen.

Die Operation hat 2 Ziele: erstens die Diagnostik und Therapie einer eventuellen PLF bzw. FAF und zweitens die Applikation von Dexamethasonphosphat möglichst nahe am runden und/oder ovalen Fenster [26].

Um den Nutzen des operativen Vorgehens zu klären, untersucht die vorliegende retrospektive Studie die tonschwellenaudiometrisch gemessene Hörverbesserung der mit einer Abdeckung des runden bzw. ovalen Fensters therapierten Patienten im postoperativen zeitlichen Verlauf. Die Beurteilung des möglichen Nutzens einer additiven Applikation von Dexamethasonphosphat am ovalen Fenster erfolgt durch eine hypothesengenerierende Subgruppenanalyse.

## Methoden und Patienten

### Ein- und Ausschlusskriterien

Anhand der Kombination der OPS-Codes 5‑202.5 (Tympanotomie und Abdeckung des runden Fensters) sowie der ICD-10-Codes H91.2 (Hörsturz) bzw. H61.1 (Perilymphfistel) erfolgte die Durchsicht aller an den HNO-Kliniken der Charité behandelten Patienten im Zeitraum 2010–2017. In die Studie wurden nur volljährige Patienten, deren präoperativer durchschnittlicher Hörverlust über 4 Frequenzen (500 Hz, 1000 Hz, 2000 Hz und 4000 Hz) mindestens >80 dB (WHO-Klassifikation) betrug, eingeschlossen.

Ausschlusskriterien waren fehlende Kontrollaudiogramme für die 2 Kontrollzeiträume, eine bilaterale Schallempfindungsschwerhörigkeit, andere Therapien und eine fehlende Bildgebung (CT Felsenbein oder MRT Kopf).

### Konservative Vorbehandlung

Alle Patienten hatten vor der operativen Therapie eine Hochdosissteroidtherapie (gemäß AWMF-Leitlinie „Hörsturz“ [2]) erhalten, beginnend mit hochdosiertem Prednisolon 250 mg i.v. Bei den Patienten der Jahre 2010–2012 erfolgte ergänzend eine Behandlung mit HAES-Infusionen (Hydroxyethylstärkelösung 6 %).

Wenn sich nach der konservativen Behandlung weder klinisch noch tonschwellenaudiometrisch eine relevante Hörverbesserung zeigte und die akute Hörminderung mindestens 3 Tage bestand, wurde die Indikation zur explorativen Tympanotomie und Rundfenstermembranabdeckung gestellt. Die Operation wurde dann als Notfall mit aufgeschobener Dringlichkeit innerhalb der folgenden 48 h durchgeführt.

### Operatives Verfahren

Der Eingriff wurde entweder in Intubationsnarkose oder in Lokalanästhesie vorgenommen. Verwendet wurde dabei nach lokaler Infiltration von 1%iger Xylocainlösung mit Adrenalinzusatz (1:200.000) ein typischer endauraler Zugang. Nach Hebung des tympanomeatalen Lappens erfolgte die Darstellung und Inspektion der Mittelohrräume, insbesondere der Rundfensternische sowie des ovalen Fensters. Zur vollständigen Einsicht wurde die knöcherne Promontoriumslippe heruntergeschliffen und ggf. dem runden Fenster vorgelagerte „falsche Schleimhautmembranen“ entfernt. Das Vorhandensein eines Flüssigkeitsspiegels als indirektes Zeichen einer PLF wurde dokumentiert. Das Wechseldruckphänomen wurde durch leichten Druck auf den Stapes getestet. Beim Zugang entnommene Temporalmuskelfaszie bzw. temporales Fettgewebe wurde in einer Lösung von ca. 0,3 ml Dexamethasonphosphat (Fortecortin® 4 mg/ml, Fa. Merck, Darmstadt, Deutschland) getränkt. Hiervon wurden kleine, ca. 1–2 mm durchmessende Gewebsstücke in die Rundfensternische eingelegt und diese damit vollständig „abgedichtet“.

Ab 2012 fand eine modifizierte Operationsmethode Anwendung. Neben dem beschriebenen Standardverfahren erfolgte zusätzlich das Einlegen von in Dexamethasonphosphatlösung getränktem Gelita im Bereich der Stapesfußplatte.

Der tympanomeatale Lappen wurde anschließend zurückgeschlagen und der Gehörgang mit in Doxycyclin getränktem Gelita tamponiert.

### Datenerhebung

Das Geschlecht, das Alter, der Zeitpunkt des Symptombeginns, der Zeitpunkt der stationären Aufnahme und die Luftleitungshörschwelle (eingeschränkter Messbereich der Knochenleitung) wurden ausgewertet. Aus den Tonschwellenaudiogrammen wurde in Anlehnung an die Empfehlungen des Committee on Hearing and Equilibrium der American Academy of Otolaryngology—Head and Neck Surgery Foundation – der Mittelwert des Hörverlusts bei den Frequenzen 500 Hz, 1000 Hz, 2000 Hz und 4000 Hz berechnet („four pure tone average“, 4‑PTA) [3]. Bei aufgrund ihrer Ausprägung nicht messbaren Hörverlusten in den Audiogrammdaten wurden bei den jeweiligen Frequenzen 120 dB HL angesetzt [7]. Der Hörverlust wurde immer in Bezug zur Normakusis und nicht in Bezug zu Voraudiogrammen oder des kontralateralen Hörbefundes ausgewertet.

Audiometrische Messungen wurden präoperativ, nach der Detamponade und als Abschlussbefund mindestens 3–6 Monate nach der Operation durchgeführt.

Zusätzlich zum Gesamtkollektiv wurden die Daten in 2 Subgruppen unterteilt und miteinander verglichen: a) Patienten, bei denen neben dem runden Fenster auch das ovale Fenster abgedeckt wurde (modifiziertes Verfahren, RF + OF), und b) Patienten, bei denen lediglich die Abdeckung der Rundfensternische erfolgte (Standardverfahren, RF).

### Hörverbesserung

Aus Mangel an internationalem Konsens zur Definition des Erfolgs einer Hörverbesserung finden bisher in den Studien verschiedene Systematiken (Siegel [33], Wilson [42], Kanzaki [12], Stachler [37], Plontke [31]) Anwendung.

Bei der Verlaufsbeurteilung der im Tonschwellenaudiogramm gemessenen Hörveränderungen wurden aufgrund von fehlenden Voraudiogrammen der betroffenen Seite die Kriterien nach Kanzaki [14] und Siegel [35] berechnet (Tab. 1, 2 und 3). Dementsprechend erfolgte die Kategorisierung in 4 Gruppen auf der Basis der durchschnittlichen Hörverbesserung im 4‑PTA bzw. 5‑PTA: Vollremission, Teilremission, leichte Remission und keine Remission.

**Table 1:** 

		*n* = 54	RF *n* = 26	RF+OF *n* = 28
Komplette Remission	Finaler Hörverlust <25 dB	1 (2 %)	1 (4 %)	0 (0 %)
Partielle Remission	Hörgewinn >15 dB Finaler Hörverlust 25–45 dB	2 (4 %)	1 (4 %)	1 (4 %)
Leichte Remission	Hörgewinn >15 dB Finaler Hörverlust >45 dB	32 (59 %)	15 (58 %)	17 (61 %)
Keine Remission	Hörgewinn <15 dB Finaler Hörverlust >75 dB	19 (35 %)	9 (35 %)	10 (36 %)

*RF* Rundfensterabdeckung,* RF+OF* Rund- und ovale Fensterabdeckung

**Table 2:** 

		*n* = 54	RF *n* = 26	RF+OF *n* = 28
Komplette Remission	Finaler Hörverlust ≤20 dB in allen Frequenzen	1 (2 %)	1 (4 %)	0 (0 %)
Partielle Remission	Hörgewinn (PTA) >30 dB	23 (43 %)	11 (42 %)	12 (43 %)
Leichte Remission	Hörgewinn (PTA) 10–30 dB	13 (24 %)	7 (27 %)	6 (21 %)
Keine Remission	Hörgewinn (PTA) <10 dB	17 (31 %)	7 (27 %)	10 (36 %)

*RF* Rundfensterabdeckung, *RF+OF* Rund- und ovale Fensterabdeckung

**Table 3:** 

		*n* = 54	RF *n* = 26	RF+OF *n* = 28
Komplette Remission	Finaler Hörverlust ≤20 dB in allen Frequenzen	0 (0 %)	0 (0 %)	0 (0 %)
Partielle Remission	Hörgewinn (PTA) >30 dB	25 (46 %)	12 (46 %)	13 (46 %)
Leichte Remission	Hörgewinn (PTA) 10–30 dB	15 (28 %)	8 (31 %)	7 (25 %)
Keine Remission	Hörgewinn (PTA) <10 dB	14 (26 %)	6 (23 %)	8 (29 %)

*RF* Rundfensterabdeckung, *RF+OF* Rund- und ovale Fensterabdeckung

### Statistische Methoden

Die statische Ergebnisdarstellung der PTA erfolgt mit Box-und-Whiskers-Grafiken. Die Boxen repräsentieren den Median und die 25- bis 75%-Quartile, der Stern den Mittelwert und die Balken die 10- bis 90%-Perzentile. Für die frequenzspezifische Darstellung der Hörverluste wurde für jede Frequenz der Median und als Maß der Streuung der Median der mittleren absoluten Abweichungen vom Median (MAD) berechnet. Sofern eine Normalverteilung der Daten vorliegt (z. B. Alter), wird der Mittelwert und die Standardabweichung angegeben. Die Prüfung auf Normalverteilung erfolgte mit dem Shapiro-Wilk-Test.

Der statistische Vergleich der prä- und postoperativen Daten erfolgte mit dem Wilcoxon-Test für gepaarte Stichproben, und für den Vergleich der unabhängigen Subgruppen wurde der Mann-Whitney-Test verwendet. Das Signifikanzniveau wurde auf 0,05 festgelegt.

## Ergebnisse

### Patientenkollektiv

**Figure 1:**
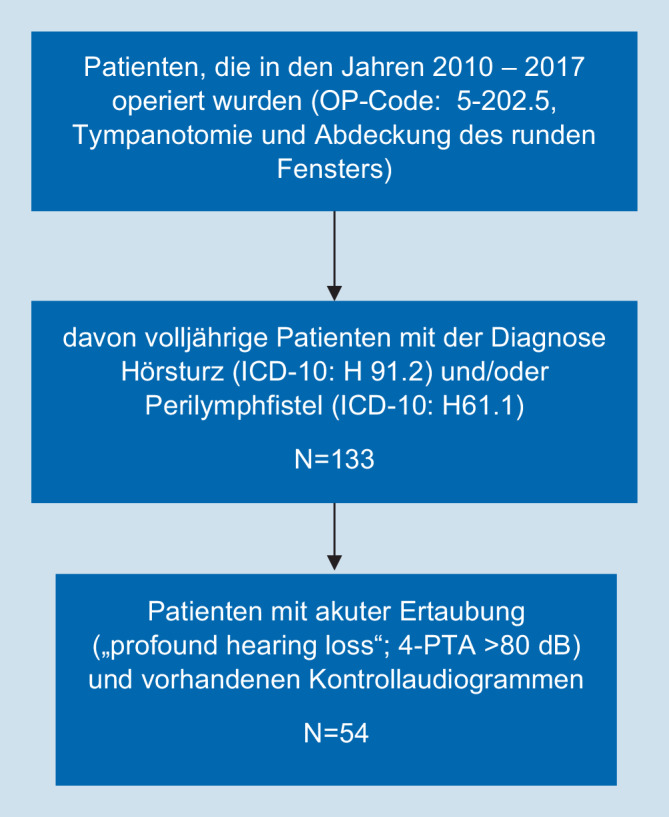

Im Zeitraum von 7,5 Jahren wurden insgesamt 133 Patienten aufgrund einer akuten hochgradigen Schallempfindungsschwerhörigkeit bzw. akuten Ertaubung tympanoskopiert. Bei 40 % der Patienten (*n* = 54) lag eine akute Ertaubung („profound hearing loss“) sowie Kontrollaudiogramme im Zeitraum von 3–6 Monaten vor. Nur diese Patienten wurden in die audiometrische Datenanalyse eingeschlossen (Abb. 1).

Von den 54 in die Studie eingeschlossenen Fällen wurden *n* = 26 mit dem Standardverfahren der alleinigen Rundfenstermembranabdeckung (RF) und *n* = 28 mit der zusätzlichen Abdeckung des ovalen Fensters (RF + OF) therapiert.

Eine intraoperativ sichtbare Ruptur der Rund- oder Ovalfenstermembran oder ein FAF wurde in keinem einzigen Operationsbericht beschrieben.

Bezüglich des Alters waren die Patienten des Gesamtkollektivs (*n* = 54) normalverteilt. Der Altersmittelwert (AMW) betrug 59 ± 17 Jahre (Spannweite 22–88 Jahre). In den Subgruppen war die Altersverteilung ähnlich (RF: AMW: 58 ± 17 Jahre, Spannweite: 29–88 Jahre; RF + OF: AMW: 63 ± 18 Jahre, Spannweite: 22–82 Jahre) Das Geschlechtsverhältnis war im Gesamtkollektiv und den Subgruppen mit 1,2:1 identisch (54 % weiblich, 46 % männlich). Das linke Ohr war bei 25 Patienten betroffen, das rechte Ohr bei 29 Patienten (RF: links *n* = 13, rechts *n* = 13; RF + OF; links *n* = 15, rechts *n* = 13).

Ein Zusammenhang der Ertaubung mit einem Baro- oder direkten Kopftrauma war bei keinem der 54 Patienten aus der Anamnese zu entnehmen. Bei 23/54 Patienten (43 %) wurden die Nebendiagnosen Herz-Kreislauf-Erkrankungen (inklusive Arterielle Hypertonie) und bei 6/54 Patienten (11 %) ein Diabetes mellitus dokumentiert, ohne diese näher zu differenzieren. Bei 17/54 Patienten (31 %) waren keine Nebendiagnosen in der Krankenakte vermerkt.

Zum Zeitpunkt der stationären Aufnahme gaben etwas mehr als die Hälfte der Patienten Schwindelsymptome an (Gesamtkollektiv: 56 %; RF: 58 %; RF + OF: 54 %).

## Therapieverlauf

Die Operation erfolgte im Median 5 Tage (Spannbreite: 3–22 Tage) nach Symptombeginn (RF: Median 5 Tage, 3–17 Tage; RF + OF: Median 4,5 Tage, 3–22 Tage). Die präoperative konventionelle Therapie in Form des Prednisolonschemas wurde im Median 3 Tage (Spannbreite: 3–19 Tage) lang angewendet (RF: Median 3 Tage, 3–10 Tage; RF + OF: Median 4,5 Tage, 3–19 Tage).

Ein erstes Kontrollaudiogramm wurde nach der Detamponade des Gehörgangs im Median am 21. postoperativen Tag (Spannbreite: 12–34 Tage) angefertigt (RF: Median 22 Tage, 12–28 Tage; RF + OF: Median 21 Tage, 14–34 Tage). Ein weiteres Kontrollaudiogramm zur Abschätzung des langfristigen Therapieerfolgs erfolgte im Median nach 4,7 Monaten (Spannbreite: 1,7–56 Monate; RF: Median 5,2 Monate; 1,7–56 Monate; RF + OF: Median 3,7 Monate; 1,4–41 Monate).

### Präoperativer Hörverlust

**Figure 2:**
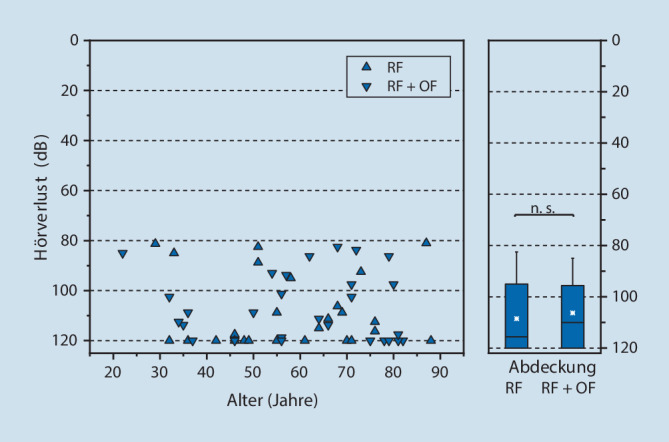

Der Hörverlust im präoperativen 4‑PTA erstreckt sich von 81 dB (HL) bis zu nicht messbaren Hörschwellen, die mit 120 dB (HL) als Zahlenwert ergänzt wurden. Der Median beträgt 113 dB (HL). Der präoperative Ausgangshörverlust weist im 4‑PTA keine Abhängigkeit vom Alter auf (Abb. 2, linke Grafik). Die Hörminderung (4-PTA) ist in beiden Behandlungsgruppen ähnlich verteilt mit RF: Median 117 dB (HL); RF + OF: Median 110 dB (HL) und weist mit *p* = 0,5 keinen statistisch signifikanten Unterschied auf (Abb. 2, rechte Grafik).

### Postoperative Veränderungen

**Figure 3:**
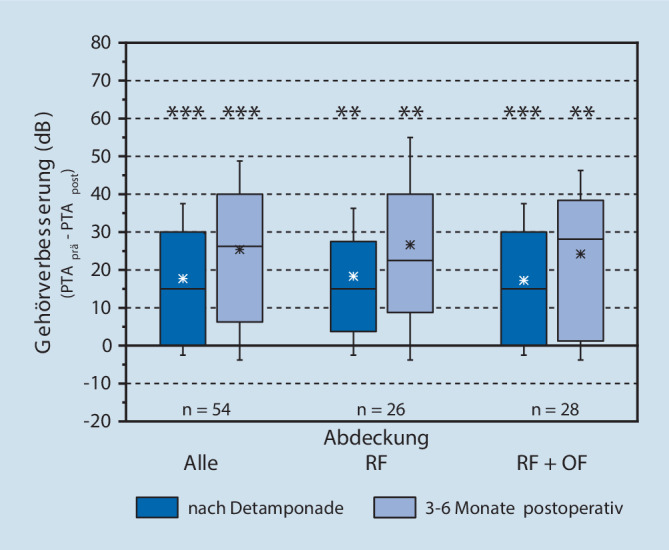

Beim Gesamtkollektiv waren die 4‑PTA-Hörschwellen (Abb. 3) im ersten postoperativen Zeitraum nach Gehörgangsdetamponade bereits hochsignifikant geringer (*p* < 0,001) und reduzierten sich im weiteren Verlauf noch mehr (3–6 Monate, gegenüber präoperativ *p* < 0,001). Die Hörverbesserung gegenüber dem präoperativen Wert betrug im ersten Kontrollzeitraum im Median 15 dB und im zweiten Zeitraum 26,3 dB (∆ = +11,3 dB). Der statistische Vergleich der Hörverbesserung zwischen den beiden postoperativen Zeiträumen weist einen hoch signifikanten Unterschied auf (*p* < 0,001).

Der gleiche Sachverhalt zeigt sich bei der Betrachtung der beiden Behandlungs-Subgruppen (Abb. 3). Sowohl in der RF-Gruppe als auch in der RF + OF-Gruppe ist die Hörschwelle im ersten Zeitraum bereits hoch signifikant geringer und in beiden Gruppen sind Median und Streuung ähnlich ausgeprägt (RF: 15 dB, *p* = 0,001; RF + OF: 15 dB, *p* > 0,001). Auch die Verbesserungen im weiteren zeitlichen Verlauf sind ähnlich (RF: ∆ = +7,5 dB, *p* = 0,002; RF + OF: ∆ = +13,1 dB, *p* = 0,003). Die im zweiten Zeitraum (3–6 Monate) im Durchschnitt um knapp 6 dB höhere Verbesserung der RF + OF-Gruppe gegenüber der RF-Gruppe ist aufgrund der starken Streuung nicht relevant. Der statistische Vergleich der Veränderungen beider Behandlungsgruppen ergibt keinen Unterschied zwischen beiden postoperativen Zeiträumen (jeweils *p* = 0,8).

Bei 9 Patienten (17 %) des Gesamtkollektivs war im zweiten Messzeitraum keine Verbesserung des PTA gegenüber dem präoperativen Zustand nachzuweisen. In der RF-Gruppe sind das nur 2/26 Patienten (7 %), wogegen es in der RF + OF-Gruppe 7/28 Patienten (25 %) betrifft.

**Figure 4:**
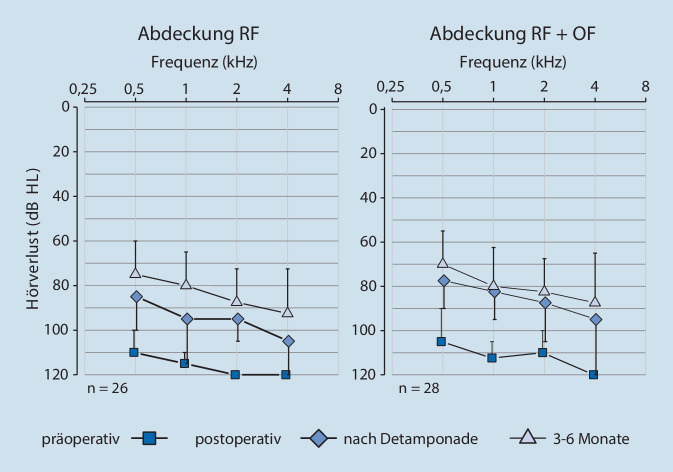

Die frequenzspezifische Detailanalyse (Abb. 4) zeigt vergleichbare Ergebnisse. Die Mediane des Hörverlusts der RF + OF-Gruppe sind in beiden postoperativen Messzeiträumen, aber auch präoperativ bei fast allen Frequenzen geringfügig besser (bis 12,5 dB) als in der RF-Gruppe. Jedoch zeigt der statistische Vergleich der Hörverluste zwischen den beiden Therapiegruppen bei den 3 Messzeitpunkten in keiner der 4 gemessenen Frequenzen (0,5; 1; 2 und 4 kHz) einen statistischen Unterschied (Mann-Whitney-U-Test: präoperativ: *p* = 0,3, *p* = 1,0, *p* = 0,3, *p* = 0,9; nach Detamponade: *p* = 0,9, *p* = 0,5, *p* = 0,3, *p* = 0,6, nach 3–6 Monaten: *p* = 0,7, *p* = 0,9, *p* = 0,7, *p* = 0,6).

Die bei den einzelnen Frequenzen gemessenen Hörverbesserungen sind innerhalb einer Behandlungsgruppe gegenüber den präoperativen Werten sowohl nach Detamponade sowie im Verlauf von 3–6 Monaten im Wilcoxon-Test jeweils auf dem Niveau *p* < 0,001 hochsignifikant.

Bezüglich der Schwindelsymptome zeigten sich auch mehrheitlich Verbesserungen der Beschwerden in den postoperativen Zeiträumen. Von den 30 Patienten mit präoperativem Schwindel klagten nach Detamponade nur noch 5 Patienten Schwindel (RF: 2; RF + OF: 3). Bei der dritten Erhebung klagten nur noch 2 Patienten über Schwindel, wobei sich beide in der RF + OF-Gruppe befanden.

Die Hörverbesserung wurde durch Berechnung des prozentualen Ausmaßes der Remission nach Siegel [35] und nach Kanzaki [14] bewertet (Tab. 1 und 3). Es fanden sich keine signifikanten Unterschiede in den Subgruppen RF und RF+OF.

Wird die Berechnung der Kriterien von Kanzaki auf ein 4‑PTA modifiziert (Tab. 2), zeigt sich eine Abnahme der Voll‑, Teil- und leichten Remissionen von insgesamt 74 % (5-PTA) auf 69 % (4-PTA) der Patienten.

In den Gruppen der 35 Patienten mit Voll‑, Teil- und leichten Remissionen fand sich zu den 19 Patienten ohne Remission bezüglich Alter, Geschlecht, betroffener Seite, Abdeckungsmethode (RF, RF+OF), Nebendiagnosen (arterielle Hypertonie, Diabetes mellitus), dem präoperativen 4‑PTA, der zeitlichen Latenz bis zum Beginn der konservativen Therapie und der zeitlichen Latenz bis zur operativen Therapie keine signifikanten Unterschiede.

## Diskussion

Inwiefern die Durchführung einer Tympanoskopie und Rundfenstermembranabdeckung bei einer akuten Ertaubung therapeutisch wirksam ist, konnte in den bisherigen Studien nicht geklärt werden [9].

### Schwierige diagnostische Ausgangslage

Möglicherweise ist dies zum Teil in der diagnostischen Ausgangslage begründet. Präoperativ ist der klinisch eindeutige Nachweis einer PLF nicht möglich. Weder das Ausmaß der Hörminderung oder die Form des Audiogramms noch das Vorhandensein eines Spontannystagmus oder Fistelsymptoms beweisen präoperativ das Vorliegen einer PLF [23]. Ebenso wenig sind Auffälligkeiten bei der kalorischen Prüfung beweisend [41]. Hornibrook beschreibt mit dem „Side-Step-Test“ einen einfachen klinischen Hinweis auf eine PLF [10]. Eine präoperativ angefertigte hochauflösende CT des Felsenbeins, bei der z. B. Lufteinschlüsse in der Cochlea sichtbar wären, könnte ggf. auf eine PLF hinweisend sein. Diesbezüglich existieren in der Literatur keine Angaben. Gemäß Tóth et al. kann eine FAF in der hochauflösenden CT an einer knöchernen Dehiszenz vor dem ovalen Fenster erkannt werden [39].

Die Bestimmung des perilymphspezifischen Cochlin-Tomoproteins (CTP) hat sich bisher nicht als Standardverfahren etabliert [11]. Die Methode eignet sich nur bedingt zur präoperativen Diagnostik. Es müsste zur Gewinnung von Untersuchungsmaterial eine Paukenhöhlenlavage erfolgen.

Der intraoperative Nachweis einer PLF durch Inspektion kann trügerisch sein und ist somit schwierig.

### Spontanheilungsraten

In der Literatur werden bei einer akuten Hörminderung ungeklärter Ursache einschließlich partieller und kompletter Spontanheilungsraten von 32 % bis zu 68 % angeben [38, 42]. Unter Berücksichtigung der Studienfallzahlen schätzen die Autoren in einem Übersichtsartikel die durchschnittliche komplette Spontanheilung auf ca. 50 % [8]. Bekannt ist aber auch, dass die Erholungsraten vom Schweregrad des initialen Hörverlusts [17, 20] und dem Frequenzspektrum (Tieftonbereich besser als Hochtonbereich) abhängig sind [21].

Die Spontanheilung einer akuten Hörminderung kann als partielle, aber auch vollständige Remission des Hörvermögens vorliegen. Aufgrund des retrospektiven Ansatzes der Studie ohne Kontrollgruppe sind Spontanremissionen der akut ertaubten Patienten nicht beurteilbar. Die partiellen Remissionen nach medikamentöser und operativer Therapie zeigen jedoch eine signifikante Hörverbesserung (Abb. 3; Tab. 4).

**Table 4:** 

Autor/Jahr	*Anzahl n* (w:m)	Mittlerer Hörverlust	PLF	Mittlere Hörverbesserung um	Kriterien der Hörverbesserung	Komplette Remission	Partielle Remission	Leichte Remission	Keine Remission/Verschlechterung
Maier 2008 [18]	76	78–104 dB	35 %	20–40 dB	k. A.	k. A.			k. A.
Gedlicka 2009 [4]	60 (k.A)	k. A.	20 %	k. A.	Kanzaki	12 %	42 %	15 %	30 %
Haubner 2012 [7]	69 (30:39)	>50 dB definiert	19 %	k. A.	k. A.	k. A.	43 % (>20 dB)	31 % (5–20 dB)	26 %
Loader 2013 [17]	25 (12:13)	k. A.	0 %	20,4 dB alle	4‑PTA 500, 1000, 2000, 3000 Hz	32 %	20 % (5/25)	k. A.	48 %
Reinecke 2013 [35]	74	58,9 dB Röser 69,2 %	42 %	12,9 dB Röser um 18,6 %	Röser 4‑PTA 500, 1000, 2000, 3000 Hz	12,3 %	k. A.		16,4 %
Kampfner 2014 [11]	101 (28:73)	101 dB HL	10 %	21,7 dB (10d postop)	Siegel	7 %	13 %	36 %	44 %
Hoch 2015 [9]	51 (32:19)	73,3 dB HL	0 %	32,7 dB	Kanzaki	23,5 %	39,2 %	15,7 %	21,6 %
Hofmann 2020 (vorliegende Studie)	54 (29:25)	107 ± 13,8 dB HL	0 %	26,3 dB	Kanzaki	0 %	46 %	28 %	26 %
					Siegel	2 %	4 %	59 %	35 %

*PLF* Perilymphfistel, *k.* *A.* keine Angaben

### Existenz der PLF

Bezüglich der intraoperativ detektierten PLF wird über eine Spannbreite von 0–60 % berichtet [1, 4, 7, 9, 12, 13, 18, 19, 33, 34, 40]. Arndt [1] fand bei 60 % (*n* = 45) und Reinecke [34] bei 42 % (*n* = 74) der Patienten intraoperativ eine PLF. In der vorliegenden Untersuchung konnte möglicherweise auch aufgrund der bedingten Einsicht (knöcherne Promontorialüberhang wurde nicht konsequent reduziert) bei keinem der 54 Patienten eine PLF oder FAF nachgewiesen werden. In 2 aktuelleren Studien mit größeren Fallzahlen bestätigte sich eine PLF intraoperativ bei 2 % der Patienten (*n* = 101) [12] bzw. gar nicht (*n* = 51) [9].

### Wirksamkeit lokal hochdosiert applizierter Kortikosteroide

Aus der Literatur lassen sich Hinweise auf die Wirksamkeit lokal hochdosiert applizierter Kortikosteroide ableiten [30], weshalb eine Tympanoskopie auch unabhängig von einem stattgehabten Trauma sinnvoll sein könnte. In Anlehnung an die positiven Ergebnisse der intratympanalen Glukokortikoidapplikation beim Hörsturz als Sekundärtherapie kann eine Tympanoskopie zum Ausschluss einer PLF und zur Applikation von dexamethasonphosphatgetränktem Bindegewebe und/oder Gelita bei hochgradigen sensorineuralen Hörminderungen (als Ultima ratio) durchgeführt werden. Jenseits der mechanistischen Vorstellung der Abdichtung könnte ein operativer Eingriff dadurch wirksam sein, dass das Medikament in hoher Dosierung an die anatomisch nächste Position zu den Innenohröffnungen (rundes und ovales Fenster) gebracht wird. Zumindest im Tierversuch konnte eine Diffusion von Medikamenten durch diese Membranen nachgewiesen werden [6, 22]. Nachdem sich bei 26 operierten Patienten (RF-Subgruppe) in keiner Operation eine FAF oder eine PLF gezeigt hatte, verlagerte sich der Schwerpunkt der bisher primär diagnostisch motivierten Operation zur therapeutischen Intervention. Dabei stellte die Rationale für die Methodenumstellung ab 2012 dar: Dexamethasonphosphatapplikation in hoher Konzentration nahe an das runde und ovale Fenster (RF + OF-Subgruppe, *n* = 28). Andere Autoren geben zu bedenken, dass die gleichzeitige Versiegelung von rundem und ovalem Fenster mit Bindegewebe zu einer bleibenden Schallleitungsstörung führen könnte [25].

### Was sagt die AWMF-Leitlinie „Hörsturz“?

Die AWMF-Leitlinie „Hörsturz“ [2] empfiehlt beim Hörsturz die intratympanale Glukokortikoidinjektion als Sekundärtherapie. Die Tympanoskopie wird in der Leitlinie als diagnostischer Eingriff erwähnt, der im Einzelfall (bei einer PLF) zu einer nützlichen Therapie werden kann.

Der Ausschluss einer retrocochleären Ursache der Hörstörung muss unbedingt vor dem Eingriff erfolgen, um eine unnötige Operation zu vermeiden. Plontke et al. konnten bei 4 % der „Hörsturzpatienten“ mittels Magnetresonanztomographie (MRT) des Schädels und des Felsenbeins ein Vestibularisschwannom nachweisen [28]. Ebenso müssen andere schwerwiegende Erkrankungen, bei denen die Hörminderung nur ein Symptom darstellt, sicher ausgeschlossen werden.

Alle hier eingeschlossenen Patienten erhielten präoperativ eine Bildgebung, in einigen Fällen wegen der Lärmbelastung bzw. Verfügbarkeit aber keine MRT, sondern nur eine hochauflösende Felsenbein-CT mit der Fragestellung nach einem Vestibularisschwannom. Mit der CT-Untersuchung können kleine Vestibularisschwannome nicht ausreichend sicher ausgeschlossen werden, sodass in der hier vorliegenden Studie eine zusätzliche Unsicherheit bleibt. Im Hinblick auf die Existenz einer traumatisch entstandenen PLF oder einer FAF wäre die präoperative Anfertigung einer hochauflösenden Felsenbein-CT zu fordern [39].

### Zeitpunkt der Operation

Auch der Zeitpunkt der Operation wird in der Literatur kontrovers diskutiert: Zunächst wird im Sinne eines Eilfalles gemäß der Leitlinie Hörsturz konservativ behandelt. Wie lange bei fehlender Besserung abgewartet werden kann bzw. abgewartet werden muss, ist unklar. Das Vorliegen einer echten PLF wäre ein Argument für eine möglichst zügige Abdichtung. Bei angenommenen kompletten Spontanheilungsraten von ca. 40–50 % im Zeitraum von Wochen [8] könnte andererseits auch eine abwartende Haltung favorisiert werden. Grundsätzlich wird ebenfalls diskutiert, ob eine Operation die Heilung theoretisch auch beeinträchtigen könnte [36].

## Operationstechnik

Die Operationstechnik betreffend existiert in der Literatur keine systematische Untersuchung unterschiedlicher Methoden. Hoch et al. [6] sowie Kampfner et al. [18] beschreiben im Methodenteil lediglich die Abdeckung des runden Fensters, während Loader et al. [18] sowohl die Abdeckung des runden als auch des ovalen Fensters dokumentieren. Im Besonderen lässt sich keine systematische Arbeit darüber finden, ob die zusätzliche Abdeckung des ovalen Fensters einen Vorteil bringen könnte.

### Hörverbesserung

Bei der Beurteilung der Hörerholung kommen in der Literatur unterschiedliche Kriterien zur Anwendung. Neben einer Abstufung des Ausmaßes der Hörerholung von einer Voll‑, Teil- und geringen Remission des Hörvermögens werden von den Autoren die 4‑PTA und auch 5‑PTA mit unterschiedlichen Frequenzen zugrunde gelegt. Das Sprachverstehen mit oder ohne Störgeräusch, als optimaler Parameter zur Beurteilung der Hörverständlichkeit im Alltag, wurde bislang in den Studien zur Tympanoskopie nicht erfasst.

In der Tab. 4 wurden die Ergebnisse der Hörverbesserung aller 54 Patienten in der vorliegenden Studie für die am häufigsten beschriebenen Kriterien von Siegel (4-PTA) [35] und Kanzaki (5-PTA) [14] den Ergebnissen anderer Autoren gegenübergestellt. Die Auswertung der eigenen Daten nach diesen beiden Kriterien ergab sehr unterschiedliche Verteilungen der Remissionsraten. Dies verdeutlicht die extrem unterschiedliche Aussagekraft und nur geringe Vergleichbarkeit dieser Studien. Grundsätzliche Limitationen sind die fehlende Kenntnis über die Hörschwelle vor der akuten Hörminderung.

In allen retrospektiven Studien (Tab. 4) erfolgte kein Vergleich zu einer Kontrollgruppe, sodass auch keine Aussage zu Spontanremissionen bei diesen höhergradigen Hörstörungen abgeleitet werden kann. Des Weiteren ist der mittlere Hörverlust präoperativ nicht immer vergleichbar. In einigen bereits zu dieser Frage erschienenen Publikationen wurden Patienten mit weniger ausgeprägten Hörstürzen eingeschlossen: Bei Reineke et al. [36] betrug der mittlere Hörverlust 58,9 dB und bei Ul-Mulk et al. [42] 67 dB vor der Operation. Dagegen wurden in der vorliegenden Studie nur Patienten berücksichtigt, die nach WHO-Kriterien an einer Ertaubung („profound hearing loss“) litten [2]. Der durchschnittliche Hörverlust im 4‑PTA betrug 107 ± 13,8 dB.

## Kritische Würdigung der eigenen Studie

Zusammenfassend kann auch die vorliegende Studie die Frage nach dem Stellenwert der explorativen Tympanotomie nicht beantworten. Die erzielten Hörverbesserungen sind nicht mit den in Metaanalysen publizierten kompletten Spontanremissionsraten von ca. 50 % [8] vergleichbar. Ursächlich dafür ist wahrscheinlich das Ausmaß der Hörstörung (hochgradige Hörstörung, Ertaubung).

Die vorliegende Arbeit hat wesentliche Limitationen. Sie beschreibt den Vergleich zweier unterschiedlicher operativen Vorgehensweisen. Auf der Basis der geringen Fallzahl von insgesamt nur 54 Patienten ergab sich statistisch kein Unterschied zwischen den Therapiegruppen RF bzw. OF/OF. Die statistische Aussagekraft ist bei der verfügbaren geringen Stichprobengröße und der starken Streuung sehr begrenzt. Mit der Annahme, dass ein Unterschied der Hörschwellenverbesserung von mindestens 10 dB als therapeutischer Unterschied zu erwarten wäre (Effektgröße ca. 0,5), resultiert mit der hiesigen Patientenzahl und ungepaarten Stichproben eine Power von nur 0,05 (α-Fehler = 0,5; ohne α‑Kumulierung). Um so einen möglichen Effekt nachzuweisen, sind Stichprobengrößen von größer *n* = 50 je Gruppe nötig.

Neben der geringen Gruppengröße, dem retrospektiven Design und der damit fehlenden Randomisierung wurden die beiden Patientengruppen zeitlich nacheinander operiert. Es zeigte sich eine erhebliche Ausfallrate. Von den initial 133 operierten Patienten lagen nur bei 54 Patienten sowohl eine echte Ertaubung („profound hearing loss“) als auch alle Verlaufs-Kontrollaudiogramme vor, d. h. der „Drop-out“ betrug 60 %.

Bezüglich der präoperativen differenzialdiagnostischen Abklärung gibt es Limitationen. Möglicherweise waren nicht alle vorliegenden MRT-Untersuchungen aus heutiger Sicht qualitativ ausreichend, um ggf. ein kleines intracochleär gelegenes Vestibularisschwannom auszuschließen [29]. Auch das Syndrom des erweiterten Aquaeductus vestibuli (LVAS) sowie der seltene Fall eines isoliert durch Hörminderung auffallenden AICA-Infarkts [16], die nicht diagnostiziert wurden, sind zu erwähnen [27].

Eine systematische Vestibularisprüfung bzw. deren Dokumentation lag nicht bei allen Patienten vor.

Die Operationen wurden von verschiedenen Operateuren vorgenommen. Inwiefern tatsächlich von allen Operateuren die Promontoriallippe zur vollständigen Einsicht des runden Fensters heruntergeschliffen wurde und „falsche Membranen“ entfernt wurden ist den Operationsberichten nicht immer explizit zu entnehmen. Dies könnte ein Grund dafür sein, dass sich bei den von 2010–2012 operierten Patienten in keinem einzigen Fall eine im Operationsbericht beschriebene PLF zeigte. Eine FAF wurde ebenfalls in keinem Operationsbericht erwähnt. Diese wurde erst 2016 in der Literatur beschrieben [39].

Eine weitere Limitation besteht in der Variabilität der Ausgangs-Tonschwellenaudiogramme der beiden Subgruppen RF- bzw. RF- und OF-Abdeckung. Bei ertaubten Patienten mit nichtmessbaren Hörschwellen wurde jeweils 120 dB HL Hörverlust angesetzt. Es kann statistisch nicht sicher ausgeschlossen werden, dass in diesen Subgruppen möglicherweise Patienten mit jeweils unterschiedlicher Qualität der Erkrankung operiert wurden.

Der Effekt der weiteren Hörverbesserung zwischen dem Zeitpunkt „nach Detamponade“ sowie „3–6 Monate postoperativ“ ist bekannt [31]. Vermutlich liegt bei der Detamponade noch eine geringe zusätzliche Schalleitungskomponente vor, die nach Ausheilung 4 Wochen später nicht mehr nachweisbar ist.

Ein Defizit dieser retrospektiven Studie ist das Fehlen einer Kontrollgruppe. Diese würde das Ausmaß der Spontanremissionen erfassen. Um eine statistisch signifikante Aussage zum klinischen Wert einer zusätzlichen Abdeckung des ovalen Fensters zu behalten, muss die Datenbasis auf eine vergleichbare Anzahl von Patienten mit akuter Ertaubung erweitert werden.

Grundsätzlich ist zur Generierung einer solchen Datenbasis zu empfehlen, dass, in Anlehnung an die HODOKORT-Studie [31], eine multizentrische, idealerweise bundesweit durchgeführte randomisierte Studie „Tympanoskopie und Rundfenstermembranabdeckung bei akuter Ertaubung“ initiiert werden sollte. Dabei könnte neben der Remission des Hörvermögens auch eine Teilremission mit ggf. durch Hörhilfen nutzbarem Resthörvermögen ein sinnvoller Endpunkt sein.

## Fazit für die Praxis

- Bei akuter Ertaubung kann eine diagnostische Tympanoskopie nach frustraner intravenöser Glukokortikoidapplikation als „Second-Line-“ sowie nach frustraner intratympanaler Glukokortikoidapplikation als „Third-Line-Behandlung“ erwogen werden.
- Es bedarf eines erfahrenen Mittelohrchirurgen, um die wichtigsten Differenzialdiagnosen PLF sowie FAF zu erkennen. Die additive Abdeckung des ovalen Fensters hat in dieser Untersuchung im Vergleich zur Abdeckung des runden Fensters zu keinem zusätzlichen Hörgewinn geführt.
- Es bedarf einer prospektiven multizentrischen Studie mit definierten Einschlusskriterien und Endpunkten, um die Wertigkeit der Tympanoskopie mit Rundfenstermembranabdeckung bei akuter hochgradiger idiopathischer sensorineuraler Schwerhörigkeit endgültig zu bewerten.

## Abkürzungen

MAD: Mediane absolute Abweichung
MW: Mittelwert
OF: Ovales Fenster
PLF: Perilymphfistel
PTA: „Pure tone average“
RF: Rundes Fenster
Spw: Spannweite
